# 

**Published:** 1885-06

**Authors:** 


PAGE.

ORIGINAL COMMUNICATIONS:

Treatment of Croup. F. E. Waxham, M. D....... 475

Peroxide of Hydrogen as a Medicinal Agent. B. Bettman, M. D....... 485

Treatment of Eczema. H. J. Reynolds, M. D.... 492

EDITORIALS:

Illinois State Medical Society....... 500

Operation for Ruptured Sac in Extra-Uterine Pregnancy....... 501

SOCIETY PROCEEDINGS:

Transactions of the Chicago Gynecological Society, April 19th....... 504

PAGE.

Thirty-fifth Annual Meeting of the Illinois State Medical Society....... 515

College of Physicians of Philadelphia, May 6, 1885....... 534

BOOK REVIEWS....... 552, 561

BOOKS RECEIVED....... 552–561

PAMPHLETS RECEIVED....... 563

LOCAL NEWS....... 564

INDEX TO VOLUME 50.

